# A meaningful, passionate life: Colleagues, friends, and family celebrate the life of Moses Schanfield (1944-2021)

**DOI:** 10.3325/cmj.2022.63.221

**Published:** 2022-06

**Authors:** Michael M. Baden, Mitchell M. Holland, Henry Lee, Haskell Pitluck, Daniele Podini, Dragan Primorac, Amanda Schwartz, Stanimir Vuk-Pavlović

**Affiliations:** 1 Former Chief Medical Examiner, New York City: Former Chief Forensic Pathologist, New York State Police, New York, New York, USA; 2 Professor of Biochemistry, Molecular Biology and Forensic Science, Pennsylvania State University, University Park, Pennsylvania, USA; 3 Professor Emeritus & Vice President and Director, Forensic Research & Training Center; Henry Lee Institute of Forensic Science, University New Haven, New Haven, Connecticut, USA; 4 Circuit Court Judge, State of Illinois (Retired); Past President, American Academy of Forensic Sciences; Past Member, Board of Illinois Judges Association; Advisor and Past Officer, Council of Scientific Society Presidents; 5 Professor of Forensic Science, The George Washington University, Washington, DC, USA; 6 Professor of Pediatrics and Forensic Science, University of Split, Split, Croatia; Pennsylvania State University, University Park, Pennsylvania, USA; President and CEO, St. Catherine Specialty Hospital, Zagreb, Croatia; 7 Judaism Your Way, Denver, Colorado, USA; 8 Professor Emeritus, Mayo Clinic College of Medicine and Science, Mayo Clinic, Rochester, Minnesota, USA (who compiled and edited this text; address correspondence to *vuk@mayo.edu*)

**Figure 1:**
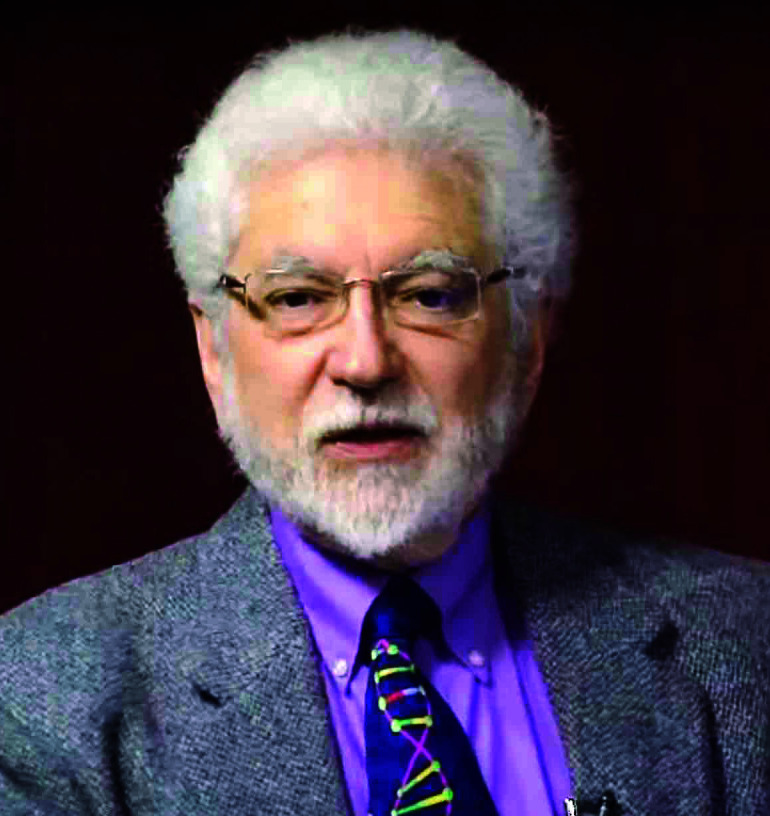
Moses Schanfield in 2015 at the 9th ISABS Conference, Bol, Island of Brač, Croatia. Photo © International Society of Applied Biological Sciences.

On the first anniversary of the passing of Professor Moses Schanfield (Figure 1), a pioneer of DNA technology in applied biology and the co-founder and co-director of ISABS Conferences (Box 1), his colleagues, friends, and daughter virtually shared their memories of the beloved friend, colleague, and father.

**Stanimir Vuk-Pavlović:** “The world is poorer for a *mensch*,” I thought reading the message that Moses died hours earlier. In my mind’s eye, I saw Moses again just like the first time – his white mane in front of the Split cathedral. Surrounded by students of the European-American Intensive Course in PCR Based Clinical and Forensic Testing, he was relishing being in their midst just as he relished everything that included science, youth, and zeal for life. It was September 23, 1997.

**Dragan Primorac:** In the fall of 1994, Dr. Henry Lee led a group to Taiwan. Both Moses and I were members of the group, and this is how we met for the first time. We befriended immediately and continued meeting at the annual conventions of the American Academy of Forensic Sciences (AAFS). Our relationship strengthened during the collaboration in identifying victims of the war of Yugoslav disintegration (1991-1995). Moses’s contribution to the field resulted in our joint pioneering publication demonstrating successful identification of mass-grave remains by DNA technology (J Forensic Sci. 41(5): 891-894, 1996).

**Henry Lee:** Moses was an innovator, scientist, colleague, and friend. I met him first in 1980, when Bob Gaensslen and I were putting out a newsletter in serology. Moses was developing the forensic use of Gm-Km markers. We spent many hours talking about it. Through our shared love of biochemistry, immunology, and forensics, we became friends quickly. We worked together on difficult cases such as a 27-year-old cold case, the murder of a young woman in New Haven. When Moses moved to Denver, he founded the Analytical Genetic Testing Center, Inc. The Center was at the forefront of developing forensic DNA standards. I made sure that I saw him every time I went to Denver; he always contacted me when he came East. Then we talked about life, family, and work – always over good Chinese food!

**Stanimir Vuk-Pavlović:** The war in the former Yugoslavia in the 1990s required advanced DNA technology that promised to greatly aid in identification of remains in mass graves in Croatia and Bosnia and Herzegovina. I was privileged to facilitate equipping of the first DNA lab in the region but that was far from enough. Moses clearly saw that the effort needed much more than just equipment. It needed local expertise; developing local expertise required a sustained educational effort.

**Dragan Primorac:** At the AAFS meeting in February 1997, Moses discussed the need to increase accessibility to scientific events for participants from outside of the United States. I suggested organizing an event in Croatia. “When would you do it?”, Moses asked. “In September!” “September, which year?” “This year,” I retorted. For years Moses would retell the event as an anecdote. At that time, the new building of the Medical School in Split was freshly completed, and it was selected for the venue of the First European-American Intensive Course held from September 23 to October 3, 1997 (https://isabs.hr/services-view/hendrerit-mauris/). From the very beginning, we included both the forensic and clinical aspects of applied molecular biology into the curricula of the meetings.

**Stanimir Vuk-Pavlović:** Moses was the *spiritus movens* of the European-American Intensive Course. Together with Dragan he organized it and led it. It was a great success that inaugurated the biannual series of the International Society of Applied Biological Sciences conferences and ISABS itself. This was just one of many things that Moses pioneered. From the very beginning, Moses set the highest instructional and scientific standards for ISABS conferences.

**Dragan Primorac:** For Moses, ISABS conferences were much more than just scientific events. He initiated and led “Meet the Professor” programs to allow young participants to mingle with senior scientists, including Nobel Prize winners; the programs were received more than well, and Moses truly enjoyed his role there. To fill the perceived need for a textbook of forensic molecular biology and a reference book for its practitioners, Moses and I led the creation of the *Forensic DNA Applications: An Interdisciplinary Perspective.* It was a privilege to participate in the project and to have Moses in this endeavor, such as in so many others, as a friend and teacher. Future editions of the book will carry this part of Moses’s legacy into the future.

**Amanda Schwartz:** My dad craved knowledge. To anyone who ever went with him to a museum or just passed by a historical marker, he had to read every word to learn every piece of information. It was the same curiosity that led to his love of travel. When I was ten, he went on a work trip to Alaska and took me along. We fished salmon, visited a husky farm, and went on a train to Mt. McKinley. That day was my birthday. Dad convinced the train conductor to lead the entire train in singing me “Happy Birthday.” When I was in high school, I told him I wanted to visit the set of the movie “Return to the Blue Lagoon.” He found the location and off we went to Fiji where we snorkeled, parasailed, and rode horses.

**Dragan Primorac:** Moses gave me advice that truly helped me making important life decisions. I miss our discussions, his wisdom, his fatherly attitude. It is difficult to accept his absence from ISABS Conferences.

**Haskell Pitluck:** I met Moses for the first time on a forensic trip to Taiwan led by Dr Henry Lee. Moses exuded competence bolstered by his accomplishments in his Colorado business. When he moved to Washington, DC our meetings continued. Then he was involved with the American Academy of Forensic Sciences in organizing Croatian meetings where he supported Dr. Dragan Primorac. During one of the meetings, overlooking the Dubrovnik Old Town, we celebrated Moses’s birthday most memorably, one can say epically. Moses and I shared good times and delicious meals while meeting interesting people. I miss Moses and cherish memories of my time with him.

**Mitchel Holland:** What I'll remember most about Moses was his smile, laughter, and wry humor. We sat together on countless occasions talking about science, human interest, and families. By the time we were heading off to the next meeting or talk, I had learned something new from him, we had laughed together, and shared a Croatian-style hug. With Moses departed, things will never be quite the same.

**Amanda Schwartz:** Dad told me once that he hoped to stay in academic life for twenty years. He was so very close to having the dream come true.

**Henry Lee:** I believe Moses was truly happy at the George Washington University, where he could teach future scientists, continue research, join colleagues at seminars and international meetings, and work to achieve justice through forensic science. At the George Washington University, my old friend Moses was close by, and we met often when I went to the DC for meetings and casework.

**Daniele Podini:** Moses was a great colleague, mentor, and friend. Truly passionate about forensic science, population genetics, and genetic anthropology. Very generous with knowledge and time, always available for a chat about a research idea, or to help a student understand the correct statistical approach to their data. I miss him so much!

**Amanda Schwartz:** In high school I struggled to get good grades. Once I asked Dad if he would shave off his beard if I got a 3.5 GPA. He had a beard his entire adult life, but he agreed. When I got that GPA, we had a beard shaving party where guests took turns shaving Dad’s beard and then had a delicious meal that he prepared. He loved cooking, especially for others.

**Michael Baden:** I met Moses in 1990 when he was among the first to study DNA techniques for the potential to improve our country’s criminal justice system. He voluntarily helped me investigate several difficult homicides for the New York State Police. He was a great forensic scientist, an excellent teacher and, above all, a beautiful human being. I do miss him.

**Henry Lee:** I was always proud to call Moses my friend. When he stood next to me, I always felt tall. Not because he was shorter in stature than I (which he was), but because of the man he was. If my scientific knowledge was less than his during a discussion, he never demeaned but raised me up. This is because Moses was the person who made *everyone* feel tall as he strove to make everyone better – a better person, a better scientist, and a better friend.

Box 1 **Biography of Moses Schanfield.**

**Moses Samuel Schanfield** (September 7, 1944–January 7, 2021) (Figure 1) studied anthropology at Harvard University and received the PhD degree from University of Michigan. At the time of death, he was a professor of forensic science at the George Washington University, where he taught population genetics, human genetic variation, and forensic molecular biology. At George Washington, Dr Schanfield served as department chair and as member of numerous university committees.

Dr Schanfield started his professional life in the early 1970s directing the Monroe County Public Safety Laboratory in Rochester, NY. Later he founded and directed the Analytical Genetic Testing Center, Inc. in Denver, CO; it was a private forensic laboratory on the forefront of developing DNA technology for forensic science. He was an expert in immune allotyping and among the first to introduce DNA analysis into parentage analysis. His laboratory helped to develop the first standard for forensic DNA testing for the National Institute of Standards and Technology of the United States. He was noted for extensive work on identification of victims of the Balkan wars of the 1990s.

Dr Schanfield authored more than a hundred peer-reviewed scientific papers, contributed forty-five book and encyclopedia chapters, and edited three books on forensic DNA testing, paternity testing, anthropological genetics, immunogenetics, and genetic basis of susceptibility to disease. As expert witness he testified in more than a hundred cases in state, federal, and military courts in the United States, Barbados, Canada, and Puerto Rico. In this capacity, he was known for the ability to disagree without being disagreeable.

For the discovery of the role of immunoglobulin subclasses in hemolytic disease of the newborn, Dr Schanfield received the Gold Medal at the First Latin American Congress of Hemotherapy and Immunohematology. He was awarded the 2021 Paul Kirk award, the highest award by the Criminalistics Section of the AAFS. He passed away a month before he could receive it in person.

